# 2-(Propyloxy)ethyl acetate: MAK Value Documentation, addendum – Translation of the German version from 2024

**DOI:** 10.34865/mb280730e11_1ad

**Published:** 2026-03-30

**Authors:** Andrea Hartwig, MAK Commission

**Affiliations:** 1 Institute of Applied Biosciences, Department of Food Chemistry and Toxicology, Karlsruhe Institute of Technology (KIT) Adenauerring 20a, Building 50.41 76131 Karlsruhe Germany; 2 Permanent Senate Commission for the Investigation of Health Hazards of Chemical Compounds in the Work Area, Deutsche Forschungsgemeinschaft, Kennedyallee 40, 53175 Bonn, Germany. Further information: Permanent Senate Commission for the Investigation of Health Hazards of Chemical Compounds in the Work Area | DFG

**Keywords:** 2-(propyloxy)ethyl acetate, irritation, haematotoxicity, MAK value, maximum workplace concentration, peak limitation, skin absorption, read-across, developmental toxicity, Luft, air

## Abstract

The German Senate Commission for the Investigation of Health Hazards of Chemical Compounds in the Work Area (MAK Commission) re-evaluated the occupational exposure limit value (maximum concentration at the workplace, MAK value) for 2-(propyloxy)ethyl acetate (2-propoxyethyl acetate) [20706-25-6] considering all toxicological end points. 2-(Propyloxy)ethyl acetate is a haemolytic and irritant glycol ether acetate. Relevant studies were identified from a literature search. The haemolytic activity of 2-(propyloxy)ethyl acetate in vivo and data from other glycol ether acetates suggest that it is probably metabolized to 2-(propyloxy)ethanol. The haemolytic activity of both compounds is mediated by the corresponding alkoxy acid; in vitro, it was lower in human erythrocytes than in rat erythrocytes. The critical effect identified in a subchronic study in rats was irritation. A NOAEC (no observed adverse effect concentration) was not obtained. The systemic NOAEC relevant for humans is 211 ml/m^3^. In analogy to 2-(propyloxy)ethanol, the MAK value of 2-(propyloxy)ethyl acetate has been lowered to 10 ml/m^3^. This exposure limit protects workers also against systemic toxicity. The substance remains assigned to Peak Limitation Category I with an excursion factor of 2. The Commission has re-evaluated a prenatal toxicity study in rats, deriving a NOAEC for developmental toxicity of 200 ml/m^3^. The margin between the NOAEC and the MAK value is sufficient even after considering the increased respiratory volume at the workplace. Therefore, damage to the embryo or foetus is unlikely if the MAK value is not exceeded and 2-(propyloxy)ethyl acetate remains assigned to Pregnancy Risk Group C. Studies investigating the genotoxic and carcinogenic potential are not available. Percutaneous absorption can contribute significantly to systemic toxicity and 2-(propyloxy)ethyl acetate remains designated with the “H” notation. In a screening study with guinea pigs, no evidence of a skin sensitizing potential was observed.

**Table:** 

**MAK value (2023)**	**10 ml/m^3^ (ppm) ≙ 61 mg/m^3^**
**Peak limitation (2000)**	**Category I, excursion factor 2**
	
**Absorption through the skin (1996)**	**H**
**Sensitization**	**–**
**Carcinogenicity**	**–**
**Prenatal toxicity (1996)**	**Pregnancy Risk Group C**
**Germ cell mutagenicity**	**–**
	
**BAT value**	**–**
	
Synonyms	ethylene glycol mono-*n*-propyl ether acetate ethylene glycol monopropyl ether acetate
Chemical name (IUPAC)	2-propoxyethyl acetate
CAS number	20706-25-6; 120519-46-2; 93215-71-5
Structural formula	CH_3_COO–CH_2_CH_2_–O–CH_2_CH_2_CH_3_
Molecular formula	C_7_H_14_O_3_
Molar mass	146.19 g/mol
Melting point	< –45 °C (Eastman Kodak Co 1982)
Boiling point at 1013 hPa	173.6 °C (Greim 1999)
Density	0.93 g/cm^3^ (Eastman Kodak Co 1982)
Vapour pressure at 25 °C	0.67 hPa (Eastman Kodak Co 1982)
log K_OW_	0.9 (XLogP3-AA; calculated) (NCBI 2022) 1.08 (calculated) (ChemSpider 2022)
Solubility	9410 mg/l water (calculated) (ChemSpider 2022) 29.17 g/l water (calculated) (Cheméo 2023)
**1 ml/m^3^ (ppm) ≙ 6.066 mg/m^3^**	**1 mg/m^3^ ≙ 0.165 ml/m^3^ (ppm)**
	
Hydrolytic stability	no data
Uses	no data

Note: The MAK value applies to the sum of 2-(propyloxy)ethyl acetate and 2-(propyloxy)ethanol.

For 2-(propyloxy)ethyl acetate, documentation from 1996 (Greim 1999) and a supplement on peak limitation are available (Greim 2000, available in German only).

The substance is not registered under REACH. It is currently not known to be in use.

Only few studies are available for 2-(propyloxy)ethyl acetate. However, its metabolism very quickly leads to the formation of the well-studied **2-(propyloxy)ethanol** in addition to acetic acid. The same effects are thus to be expected for 2-(propyloxy)ethyl acetate as for **2-(propyloxy)ethanol**. Therefore, the MAK value of 20 ml/m^3^, the peak limitation category and the Pregnancy Risk Group C established for **2-(propyloxy)ethanol** were adopted in 1996 (Greim 1999). In 2017, **2-(propyloxy)ethanol** was re-evaluated (Hartwig and MAK Commission 2019) and the MAK value was lowered. Therefore, also the data for 2-(propyloxy)ethyl acetate have been re-evaluated.

## 1 Toxic Effects and Mode of Action

2-(Propyloxy)ethyl acetate is slightly irritating to the skin and eyes of animals. Haematotoxicity with secondary effects on the spleen were observed in rats. These effects occurred after inhalation exposure to concentrations of 221 ml/m^3^ and above for 90 days. At concentrations of 340 ml/m^3^ and above, body weight gains were delayed. Clinical signs of irritation were observed in all groups. 2-(Propyloxy)ethyl acetate caused developmental toxicity with delays in ossification in rats at 400 ml/m^3^ and above. In a screening study in guinea pigs, 2-(propyloxy)ethyl acetate was not found to be skin sensitizing. There are no data available for genotoxic or carcinogenic effects of the substance.

## 2 Mechanism of Action

As is known for ethylene glycol ethers and their acetates, their toxicity is due to the haemolytic activity of the alkoxyacetic acids formed during metabolism. The exact mechanism of haematotoxicity is not known. Humans are significantly less sensitive to this haematotoxic effect than rats (Hartwig and MAK Commission 2019).

## 3 Toxicokinetics and Metabolism

There are no studies available for the metabolism of 2-(propyloxy)ethyl acetate. In view of the haematotoxic effect (see Sections 2 and 5.2), the metabolism of 2-(propyloxy)ethyl acetate to **2-(propyloxy)ethanol** is plausible.

The blood:air partition coefficient, calculated according to the formula of Buist et al. (2012) with log K_OW_ 0.9, is about 7300.

Male beagle dogs were exposed to 50 ml 2-(propyloxy)ethyl acetate/m^3^ for 5 hours. Pulmonary absorption at steady state after 3 hours was 74% (Guest et al. 1984).

Dermal absorption was determined in three beagle dogs. A quantity of 15 ml of undiluted radiolabelled 2-(propyl­oxy)ethyl acetate was applied occlusively to 55.6 cm² of shaved skin for 30 or 60 minutes. The uptake was calculated from the radioactivity excreted in the urine and corrected on the basis of that excreted after intravenous administration. The flux was 179.2 nmol/cm² and minute, corresponding to 1571 µg/cm² and hour (Guest et al. 1984). Under standard conditions (2000 cm², 1 hour), an amount of 3140 mg would be absorbed.

In Franz cells, 2-(propyloxy)ethyl acetate (0.3 ml, 0.9 cm²) was applied to dog skin for up to 7 hours. The receptor medium was physiological saline. The substance did not cause significant damage to the skin. The flux was 163 nmol/cm² and minute (Guest et al. 1984), which corresponds to 1.430 mg/cm^2^ and hour. Under standard conditions, 2860 mg would be absorbed. The authors investigated also **2-ethoxyethyl acetate**, for which they calculated a flux of 279.7 nmol/cm² and minute (Guest et al. 1984), which corresponds to 2.217 mg/cm^2^ and hour.

For **2-ethoxyethyl acetate**, the flux through human skin is 0.8 mg/cm^2^ and hour (Dugard et al. 1984). Dog skin thus appears significantly more permeable than human skin.

After intravenous injection of radiolabelled 2-(propyloxy)ethyl acetate in beagle dogs, 61% of the dose (1 mg/kg body weight) was excreted in the urine within 4 hours and 88% within 24 hours. The initial half-life was 1.6 hours. About 1.6% was exhaled as CO_2_, other metabolites were not determined (Guest et al. 1984).

## 4 Effects in Humans

There are no data available.

## 5 Animal Experiments and in vitro Studies

### 5.1 Acute toxicity

There are no new data available.

The 6-hour LC_50_ in rats was found to be 934 ml/m^3^, the oral LD_50_ in rats 9450 mg/kg body weight and the dermal LD_50_ in guinea pigs more than 20 000 mg/kg body weight (Greim 1999).

### 5.2 Subacute, subchronic and chronic toxicity

#### 5.2.1 Inhalation

In a 2-week study in COBS/CD rats, the NOAEC (no observed adverse effect concentration) for haemolytic activity, as the most sensitive end point, was 100 ml 2-(propyloxy)ethyl acetate/m^3^, and for all other effects 800 ml/m^3^ (highest concentration tested) (Greim 1999).

In a previously unreported 90-day study, 20 male and 20 female CRL:COBS CD(SD)BR rats were exposed whole-body to 2-(propyloxy)ethyl acetate concentrations of 0, 97, 148, 221 or 340 ml/m^3^ for 6 hours daily, on 5 days per week. In addition, 15 male and 15 female animals were likewise exposed to 0, 148 or 340 ml/m^3^ and followed up for 4 weeks. In the male animals, the incidence of polychromasia was increased at and above 148 ml/m^3^ (at 148, 221, 340 ml/m^3^: 3/20, 5/18, 11/20, respectively; no other details). At 221 ml/m^3^ and above, haematotoxic effects such as haemoglobinuria and macrocytic normochromic anaemia with secondary effects in the spleen (increased absolute and relative weights, congestion, haemosiderosis, extramedullary haematopoiesis) occurred in both sexes. The terminal body weights and body weight gains of the female animals were reduced in a statistically significant manner at 340 ml/m^3^. No other effects on clinico-chemical and urine parameters, organ weights, the histopathology of 43 tissues including nasal tissue (not further specified) or reproductive organs were observed. Clinical observations revealed signs of irritation, but these were not concentration-dependent (no other details; data for the individual animals are not included in the study report). Therefore, the authors did not consider them to be substance-related. Except for the effects on the spleen at 340 ml/m^3^, all findings were reversible. The authors reported a NOAEC of 148 ml/m^3^ (Eastman Kodak Co 1985). As humans are significantly less sensitive to haematotoxicity (see Section 2), the NOAEC for systemic effects that are relevant for humans is 221 ml/m^3^ due to delayed body weight gains at 340 ml/m^3^. The NOAEC for irritant effects is unclear.

The effect spectrum and effect strength of 2-(propyloxy)ethyl acetate are thus similar to those of **2-(propyloxy)ethanol** (Hartwig and MAK Commission 2019) with a NOAEC of 100 ml/m^3^ for haematotoxicity in a 90-day study in rats. For **2-(propyloxy)ethanol**, however, the nasal irritation in the 90-day study was more pronounced, as corresponding clinical findings occurred at the lowest concentration tested of 100 ml/m^3^.

#### 5.2.2 Oral administration

There are no new data available.

In a 6-week study, male rats were given daily doses of 1097 to 4386 mg/kg body weight by gavage. Haemolytic activity occurred in all groups, spermatotoxicity at and above 2193 mg/kg body weight and day, and damage to the liver and bone marrow and mortality at 4386 mg/kg body weight and day (Greim 1999).

#### 5.2.3 Dermal application

There are no new data available.

Skin irritation was observed after non-occlusive application of 2-(propyloxy)ethyl acetate to the dorsal skin of guinea pigs for 14 days. Systemic toxicity was not investigated (Greim 1999).

### 5.3 Local effects on skin and mucous membranes

There are no new data available.

2-(Propyloxy)ethyl acetate is slightly irritating to the skin of guinea pigs and slightly irritating to the rabbit eye (Greim 1999). The eye irritation caused by **2-(propyloxy)ethanol** is stronger than that caused by 2-(propyloxy)ethyl acetate (Hartwig and MAK Commission 2019).

### 5.4 Allergenic effects

There are no new data available.

In a screening test, the substance was not sensitizing in guinea pigs (Greim 1999).

### 5.5 Reproductive and developmental toxicity

In a prenatal developmental toxicity study carried out according to OECD Test Guideline 414, COBS CD(SD)BR rats (20 to 33 animals per group) were exposed whole-body to 2-(propyloxy)ethyl acetate concentrations of 0, 100, 200, 400 or 800 ml/m^3^ (6 hours per day; vapour) from days 6 to 15 of gestation. At concentrations of 200 ml/m^3^ and above, a statistically significant increase in the incidence of rudimentary 14^th^ thoracolumbar ribs was observed. There were also isolated cases of delayed ossification of the hyoid bone, vertebral centra (statistically significant increase on a foetal basis) and sternum, the incidence of which was not increased with statistical significance on a foetal and litter basis. At 400 ml/m^3^ and above, there were additionally delays in the ossification of the skull and vertebral centra. At 800 ml/m^3^ the frequency of additional ribs (more than half the length of the 13^th^ rib) and the average number of resorptions per litter (4.0 ± 4.7 compared with 0.95 ± 0.97 in the controls) were increased with statistical significance. The latter could be attributed to a dam with total resorption. At concentrations of 200 ml/m^3^ and above, the mean corpuscular volume of the erythrocytes increased in the dams (evidence of erythropoiesis). With increasing concentration, also haemolytic effects and haemoglobinuria, reduced food intake and delayed body weight gains occurred. The NOAEC for maternal toxicity was 100 ml/m^3^ (Greim 1999; Krasavage and Katz 1984).

The experimental data obtained in the rat for 2-(propyloxy)ethyl acetate and **2-(propyloxy)ethanol** are in good agreement. Similarly high incidences of rudimentary 14^th^ thoracolumbar ribs (a variation) occurred at the same concentration of 2-(propyloxy)ethyl acetate and **2-(propyloxy)ethanol** (see Table 1; Krasavage and Katz 1984, 1985). The statistical significance was checked retrospectively using the chi-square test. For 2-(propyloxy)ethyl acetate, at 200 ml/m^3^ there was no statistically significant difference on a foetal basis (p = 0.069) compared with the incidence in the control group, but there was a statistically significant difference on a litter basis (p = 0.041). At 25% on a foetal basis and 70% on a litter basis, the incidences of rudimentary thoracolumbar 14^th^ ribs in rats at the 2-(propyloxy)ethyl acetate concentration of 200 ml/m^3^ (Krasavage and Katz 1984) lie at the upper end of the historical control range (MARTA 1993: 1979–1992: highest incidence on a foetal basis: 25%, on a litter basis: 75%). Moreover, rudimentary 14^th^ ribs in rats is a transient change (DeSesso and Scialli 2018). Therefore, the rudimentary 14^th^ ribs in rats are not regarded as adverse. The NOAEC for developmental toxicity for the rat is 200 ml 2-(propyloxy)ethyl acetate/m^3^, as at 400 ml/m^3^ delays in ossification begin.

**Tab. 1:** Comparison of the incidence of rudimentary 14^th^ thoracolumbar ribs in rats after exposure to 2-(propyloxy)ethyl acetate (Krasavage and Katz 1984) and 2-(propyloxy)ethanol (Krasavage and Katz 1985)

**Incidence**	**2-(Propyloxy)ethyl acetate [ml/m^3^]**					
	**0**	**100**	**200**	**300**	**400**	**800**
foetuses (%) litter (%)	19/119 (16%) 8/21 (38%)	24/121 (20%) 14/21 (67%)	32/126* (25%) 14/20*^, a)^ (70%)	not tested	48/153^a)^ (31%) 18/23*^, a)^ (78%)	71/108*^, a)^ (66%) 19/19*^, a)^ (100%)
	**2-(Propyloxy)ethanol [ml/m^3^]**					
	**0**	**100**	**200**	**300**	**400**	**800**
foetuses (%) litter (%)	18/130 (14%) 9/24 (37%)	25/133 (19%) 13/25 (52%)	36/138* (26%) 17/25* (68%)	59/138* (43%) 20/24* (83%)	73/153* (48%) 21/28* (75%)	not tested

*p < 0.05 in the chi-square test, (2 × 5) table, according to the authors

^a)^ p < 0.05 in the chi-square test, (2 × 2) table, subsequent analysis

No other species was investigated with 2-(propyloxy)ethyl acetate.

**2-(Propyloxy)ethanol** did not cause any developmental toxicity in a prenatal toxicity study in New Zealand White rabbits exposed to concentrations of 0, 125, 250 or 500 ml/m^3^ for 6 hours daily from days 6 to 18 of gestation. At 500 ml/m^3^, one dam exhibited haemoglobinuria and there was a slight reduction in the body weight gains of the dams (Greim 1999). The NOAEC for developmental toxicity is therefore 500 ml **2-(propyloxy)ethanol**/m^3^ for rabbits.

### 5.6 Genotoxicity

There are no data available.

### 5.7 Carcinogenicity

There are no data available.

## 6 Manifesto (MAK value/classification)

The critical effects are irritation and haematotoxicity. However, humans are less sensitive to the latter than rats.

**MAK value. **In analogy to that for **2-(propyloxy)ethanol**, a MAK value of 10 ml/m^3^ has been set for 2-(propyl­oxy)ethyl acetate. This value protects against irritant effects as 2-(propyloxy)ethyl acetate is less severely irritating than **2-(propyloxy)ethanol**. This procedure corresponds to that for 2-butoxyethyl acetate, whose MAK value was set also in analogy to that for the more severely irritating alcohol 2-butoxyethanol (Greim 2008, available in German only).

Based on the systemic NOAEC of 221 ml/m^3^ obtained from the 90-day study, a concentration of 28 ml/m^3^ would result for exposure at the workplace (221 ml/m^3^ / 2 (data from animal experiments) / 2 (extrapolation from subchronic to chronic exposure) / 2 (increased respiratory volume at the workplace) = 28 ml/m^3^). Thus, the MAK value of 10 ml/m^3^ derived in analogy to that for **2-(propyloxy)ethanol **protects workers also against systemic effects.

**Peak limitation. **The assignment of 2-(propyloxy)ethyl acetate to Peak Limitation Category I has been retained due to its critical local effects. The excursion factor of 2, as set for **2-(propyloxy)ethanol**, likewise remains valid and was established in the supplement (Greim 2000).

**Prenatal toxicity. **2-(Propyloxy)ethyl acetate leads to a statistically significant increase in the incidence of rudimentary 14^th^ thoracolumbar ribs in rats at and above 200 ml/m^3^, which is not regarded as adverse (see Section 5.5). The NOAEC for developmental toxicity in the rat is 200 ml/m^3^, as ossification delays begin at 400 ml 2-(propyloxy)ethyl acetate/m^3^. A teratogenic effect was not observed.

**2-(Propyloxy)ethanol** was not found to be teratogenic in prenatal toxicity studies in rats and rabbits. Developmental toxicity occurred with both substances only at maternally toxic concentrations (Greim 1999; Krasavage and Katz 1984, 1985).

The animal studies were conducted with whole-body exposure. Therefore, dermal exposure to the gaseous phase is included, which corresponds to the maximum possible exposure. Taking into consideration the increased respiratory volume (1:2), the NAEC (no adverse effect concentration) for developmental toxicity is 10 times the MAK value of 10 ml/m^3^, which is a sufficient margin. The metabolite **2-(propyloxy)ethanol** is assigned to Pregnancy Risk Group C with a MAK value of 10 ml/m^3^. Assignment to Pregnancy Risk Group C has therefore been confirmed for 2-(propyl­oxy)ethyl acetate.

**Carcinogenicity. **No studies are available for this end point and there is no structural alert.

**Germ cell mutagenicity. **No studies are available for this end point and there is no structural alert.

**Absorption through the skin. **Studies in dogs in vivo and with dog skin in vitro resulted in an amount of about 3000 mg absorbed under standard conditions. The systemic NAEC extrapolated to humans is 28 ml/m^3^ (170 mg/m^3^) (see “MAK value”). At a respiratory volume of 10 m^3^ and 74% absorption by inhalation (from data for dogs (Guest et al. 1984)), this corresponds to a systemically tolerable amount of 1260 mg. The amount absorbed through the skin is thus higher than 25% of the systemically tolerable amount and the previous designation with an “H” (for substances which can be absorbed through the skin in toxicologically relevant amounts) has been retained.

**Sensitization. **There are still no studies available for the sensitizing effects of 2-(propyloxy)ethyl acetate in humans and no new results from animal experiments or from studies with alternative test methods (new approach methods). Therefore, 2-(propyloxy)ethyl acetate remains not designated with “Sh” or “Sa” (for substances which cause sensitization of the skin or airways).
